# Applying Spring Security Framework with KeyCloak-Based OAuth2 to Protect Microservice Architecture APIs: A Case Study

**DOI:** 10.3390/s22051703

**Published:** 2022-02-22

**Authors:** Ayan Chatterjee, Andreas Prinz

**Affiliations:** Department of Information and Communication Technology, Centre for e-Health, University of Agder, 4630 Kristiansand, Norway; andreas.prinz@uia.no

**Keywords:** API, REST, spring-boot, Keycloak, authentication, authorization, encryption, external attacks

## Abstract

In this study, we implemented an integrated security solution with Spring Security and Keycloak open-access platform (SSK) to secure data collection and exchange over microservice architecture application programming interfaces (APIs). The adopted solution implemented the following security features: open authorization, multi-factor authentication, identity brokering, and user management to safeguard microservice APIs. Then, we extended the security solution with a virtual private network (VPN), Blowfish and crypt (Bcrypt) hash, encryption method, API key, network firewall, and secure socket layer (SSL) to build up a digital infrastructure. To accomplish and describe the adopted SSK solution, we utilized a web engineering security method. As a case study, we designed and developed an electronic health coaching (eCoach) prototype system and hosted the system in the expanded digital secure infrastructure to collect and exchange personal health data over microservice APIs. We further described our adopted security solution’s procedural, technical, and practical considerations. We validated our SSK solution implementation by theoretical evaluation and experimental testing. We have compared the test outcomes with related studies qualitatively to determine the efficacy of the hybrid security solution in digital infrastructure. The SSK implementation and configuration in the eCoach prototype system has effectively secured its microservice APIs from an attack in all the considered scenarios with 100% accuracy. The developed digital infrastructure with SSK solution efficiently sustained a load of (≈)300 concurrent users. In addition, we have performed a qualitative comparison among the following security solutions: Spring-based security, Keycloak-based security, and their combination (our utilized hybrid security solution), where SSK showed a promising outcome.

## 1. Introduction

### 1.1. Overview and Motivation

Security in the healthcare system has been an emerging trend for the past few years. It defines the interconnection of communication-enabled medical-grade devices (e.g., wearable and non-wearable), web services, software applications, and their integration with wider-scale health systems and services to improve patients’ wellbeing [1,2]. However, the growth and widespread adoption of the health security ecosystem has benefited sensor-based remote monitoring (including wearable and stand-alone devices) with the management of personal and person-generated health data [1,2].

Articles related to eHealth security research unveil the following security and privacy requirements in healthcare systems (both on-premises and cloud-based) [1,2]: data security, user authentication, regulatory compliance, authorized access, confidentiality, ethical consent, legal issues, the relevance of data access, data ownership, data consistency, data separation, audits, archiving, third-party certificates (such as SAS70 Type II, Payment Card Industry Data Security Standard (PCI DSS) Level 1, International Organization for Standardization (ISO) 27001, and Federal Information Security Management Act (FISMA)), protection against network security issues (such as Denial-of-Service (DoS), Distributed DoS (DDoS), Man in the Middle (MITM) attack, Internet Protocol (IP) spoofing), policies, protocols, and database security. From the existing studies, identified vital security terms (see Table 1) are distributed in the following four categories to use in this study: authentication (multi-factor, form-based, bearer token, and API key), authorization (Open Authorization (OAuth2), Open Identifier (OpenID), and Cross-Origin Resource Sharing (CORS)), encryption (digital certificate, Hypertext Transfer Protocol Secure (HTTPS), Rivest–Shamir–Adleman (RSA), Bcrypt, Secure Hash Algorithm (SHA)-256, and Message-Digest algorithm (MD5)) and external security threats (Cross-Site Request Forgery (CSRF), MITM, Cross-site scripting (XSS), brute force, DoS, DDoS, and IP spoofing).

Authentication is used to verify personal identity; authentication is about validating credentials. The scheme decides whether the person is a legal consumer. Authorization is the mechanism used to determine if specific services are available to the authenticated user. It verifies users’ rights to have personal access to resources such as records, databases, and files. Typically, authorization comes after authentication to verify user privileges. Encryption is a mechanism that encodes a document or file such that only some individuals can read it. Encryption uses an algorithm to encrypt data at the sender side and a key to decrypt the encrypted data into original form at the receiver side. External threats refer to attacks from external systems using malicious software, hacking technologies, social engineering, and attempts to exploit system vulnerabilities. Healthcare research [2,3,4,5,6,7,8,9,10,11,12] shows multiple studies associated with API security, protection of Electronic Health Records (EHR), secure Internet-of-Medical-Things (IoMT) system, security protocols and authentication scheme, methods for healthcare security, healthcare cloud and big data security, healthcare security compliance, security performance, challenges, and success factors.

Salibindla et al. [13] conducted a study on microservice API security focusing on the security for communication protocols. Xie et al. [14] published a report on Spring Security architecture and its implementation. However, these studies were performed separately without verifying the efficacy of the Spring Framework (SF), SSF, and OAuth2 when these technologies were used on MSA endpoint authentication and authorization. Nguyen et al. [15] created a proof-of-concept (PoC) MSA application using SF, spring protection, and OAuth2 to reduce the information gap on MSA and API security. They did not test how the solution would work after integrating with a third-party IAM platform. Dikanski et al. [16] performed a conceptual study to identify and implement authentication and authorization patterns in the SSF to reduce the difference between design and implementation of pattern-based protection to incorporate high-quality security features in software systems. However, the study suffered from SSF’s actual implementation to protect MSA at the API endpoint level with integration with the IAM platform. Aloufi et al. [17] proposed a secure and cost-effective model based on Message Queue Telemetry Transport (MQTT) protocol to secure IoT resources with access control mechanisms over RESTful web services. Beer et al. [18] proposed a neural network-based adaptive security architecture to protect RESTful web services in an enterprise computing environment with the following three functional principles: predict, prevent, and learn an intelligent approach to detect future threats. The core of their proposed security solution was based on the public key infrastructure (PKI) and its related encryption technology to protect HTTP transactions. The results were compared with supported network/transport layer security, and it was discovered that the proposed security solution is suitable for REST APIs and is better than the Simple Object Access Protocol (SOAP)-based web services. Since RESTful web services are stateless, they usually do not have any sessions to perform challenge-response mechanisms. Transport layer security/secure socket layer (Transport Layer Security (TLS)/Secure Socket Layer (SSL)) provides secure peer-to-peer authentication. Still, when the authentication request is based on delegation, this mechanism is inadequate to allow sites to authenticate on behalf of their users. HTTP security (HTTPS) is widely used; however, it only provides hop-by-hop protection. A good solution that follows RESTful principles is the token-based approach. Serme et al. [19] proposed a security model based on confidentiality and digital signatures to protect RESTful messages. These messages carry tokens for non-repudiation and provide data secretly by encrypting their content. They proposed a protocol to ensure the communication security of RESTful services. They provided encryption, signature, and their combination. They did not intend to offer an equivalent secure session for RESTful services because it relates to the transport layer security of HTTP, which has already been addressed in protocols, such as SSL and TLS. Backere et al. [20] designed a security mechanism for RESTful web services using non-RESTful elements to outperform TLS.

However, only a few studies concentrate on implementing the Spring Security Framework (SSF) and Microservice Architecture (MSA) at the API endpoint level, regardless of the vital role of API protection in MSA with an open-source Identity and access management (IAM) platform. This has been the motivation behind conducting this study.

### 1.2. Aim of the Study

This study aims to reduce the MSA and API security knowledge gap and protect REST APIs by creating an MSA application prototype using SSK. In conjunction with open source Keycloak software (Apache license 2.0), we perform a study on spring-based API protection solutions with critical features, such as identity brokering, OAuth2, multi-factor authentication, CORS, and user management against unauthorized access and external attacks [13,14,21]. We used SHA256 hashing with ECDSA algorithm (or ES256) to create a JWT signature, pbkdf2-sha256 for password management in Keycloak, and SHA512 for the OTP hash algorithm in Keycloak. We focused on active sniffing with the Wireshark network analyzer [22]. We conducted non-functional security testing, such as penetration testing, to validate security gaps and confirm the usefulness of the open-source SSF and Keycloak in securing REST APIs. Moreover, we performed unit testing to validate the security performance for each module. The research questions for this study are as follows:

(RQ1) How to develop an integrated security solution with Spring Security framework (SSF) and open-access identity and access management platform (IAM)? How to extend the security solution to build a digital infrastructure?
(RQ2) How scalable and relevant is the adopted solution to protect Microservice Architecture APIs from external vulnerabilities?

Different open-access IAM platforms are available in the market, such as Keycloak, ADFS 2.0, Shibboleth, Open AM/OpenSSO, Ping Federate, and Okta. However, to achieve IoMT REST-API security in this study, we used the Keycloak platform and SSF (SSK). The Keycloak-based OAuth2 solution can be replaced with other IAM platform-based OAuth2 for mutual performance comparison; it is beyond the scope of this study. Subsequently, we expanded the SSK solution with VPN, Bcrypt, API key, network firewall, and SSL protocol to build a digital infrastructure to host health applications. SSK implements security features, such as identity brokering, OAuth2, multi-factor authentication, CORS, and user management to protect the REST APIs from illegitimate access and external attacks, such as CSRF, XSS, Clickjacking, content sniffing, brute force, DoS, DDoS, IP spoofing, and MITM. After deploying an electronic health coaching (eCoach) prototype system in the developed digital infrastructure, we conducted a formal security analysis of the SSK solution scheme. In Section 3 and Section 4, we explain the RQ1, and the RQ2 has been elaborated in Section 5 and Section 6.

## 2. Basic Preliminaries

In a digital health system [23,24,25,26], personal health data can be collected from heterogeneous sources over the different web and REST APIs. Therefore, maintaining the privacy and security of EHRs has become an open challenge. In combination with open source KeyCloak software [27], the Spring Security [14,15,16] paradigm may offer an opportunity to enhance security features, functionalities, identity brokering, session handling, CORS support, access management solutions, and security assertion markup language (SAML) [28] for a health system’s web and REST APIs. The required security background for understanding the SSK security solution is discussed in this section.

Spring Security Framework [14,15,16] is a robust, highly customizable, comprehensive, and extensible open-source Java framework supporting authentication and authorization. Furthermore, it provides a solution to protect common external attacks [28,29,30,31,32,33,34,35,36,37], such as session fixation, clickjacking, CSRF, etc. For securing Spring-based applications, it is the de-facto standard. The modules linked to spring security are core, remoting, web, config, LDAP, Access Control List (ACL), Central Authentication Service (CAS), and OpenID. The modular SSF consists of loosely coupled components linked to dependency injection. The selected list of core security components (class/interface) of the SSF is described in Table 2. SSF can be integrated with various authentication technologies and standards, such as single-sign-on or SSO [28] (Kerberos, LDAP, and JASSO), HTTP-basic, automatic remember-me, and form-based authentication, authentication filter, OpenID [28], OAuth [29], and SAML. This study focused mainly on form-based authentication, OAuth 2.0, SAML 2.0, standard spring security programming models, and configuration idioms. Spring Security OAuth supports using spring security with OAuth (v. 1a) and OAuth2. The spring SAML extension (SSE) enables SAML 2.0 service provider features to integrate with spring applications. It supports SAML 2.0 in identity provider (IP) mode with Keycloak, ADFS 2.0, Shibboleth, Open AM/OpenSSO, Ping Federate, and Okta.

Keycloak [27] is an open-access IAM platform that secures web applications and RESTful web services using standard protocols such as OAuth2, OpenID Link, and SAML 2.0. It offers flexible login, registration, administration, CORS support, and account management user interfaces. We configured Keycloak as a separate sever to secure our eCoach APIs using the standard security assertion markup language 2.0 (SAML 2.0) integration with SSF. SAML 2.0 is a variant of the SAML standard that allows security domains to share authentication and authorization identities. SAML 2.0 is an XML-based protocol that uses security tokens containing assertions between an SAML authority, an Identity Provider, and an SAML client, called a Service Provider, to transfer information about a principal (usually an end-user). SAML 2.0 allows SSO, web-based, cross-domain, and helps minimize the administrative overhead of transmitting multiple authentication tokens to the user. These tokens may have identity details, such as username, address, email, and other information about the profile. They can also keep permission data so that users can make authorization decisions. These tokens can also be used on REST-based services to render stable invocations. The following core concepts and terms are generally used in Keycloak to secure web application and REST APIs [23]: users, authentication, authorization, credentials, roles, user role mapping, composite roles, groups, realms (to manage a set of users, credentials, their functions, and groups), clients, client adapters, client role, identity token, access token, session, user federation provider, and identity provider mappers. Table 2 describes the selected list of components of Keycloak for SSF integration to protect our eCoach REST APIs.

Microservice Architecture (MSA) [29,30,31,32,33,34,35,36,37] has arisen to describe a specific way of developing software systems for independently deployable services. The traditional monolithic software development approach suffers from the following drawbacks: bundled deployment as a single stack, intransigent scalability, high cost of resources and refactoring efforts, and DevOps challenges among dispersed teams [15,16]. In contrast, MSA handles such concerns with the following measures: task decomposition into services, service communication using APIs with the smallest granularity, agility, independent deployment, and execution of services. REST and SOAP are two HTTP-based communication protocols used for data exchange between microservice APIs in multiple formats, such as XML and JSON [15]. We used REST for eCoach API implementation in this study due to its lightweight nature. There are numerous methods to secure REST APIs. Still, the following four are the most popular:

- HTTP-based authentication scheme (basic and bearer token),
- API keys,
- OAuth2 (access token and refresh token), and
- OpenID (e.g., Keycloak OpenID, BankID).

OAuth2 is intended for authorization only, to grant access from one program to another to data and features. OpenID Connect (OIDC) is a thin layer on top of OAuth2 that adds details about logging into the user profile. We used Keycloak generic OpenID connect relying party and SAML service provider libraries in this study. The reliability and credibility of eHealth scientific research and associated services rely on the health data protection plans and guidelines regarding security, privacy, and confidentiality. For this study, we received ethical approval from The Norwegian Centre for Research Data (NSD) for managing data for our eHealth research in Norway.

## 3. System Architecture

Personal health and wellness data are generally collected through wearable sensors, interactions, interviews, web-based interactions, mobile apps, questionnaires, and feedback forms [23,25]. For ubiquitous tracking, high-end, time-dependent activity data collection with wearable BLE devices has become accessible and feasible. Wearable activity sensors can be connected via Bluetooth short-range communication technology (BLE) to a smartphone. With a machine intelligence module, the computing device can calculate and transfer high-resolution raw acceleration data and multiple operation parameters to safe storage seamlessly to process the data further. Some activity data are questionnaire-dependent, such as non-wear time and intense activity information. Either invasive (e.g., glycemic response, cholesterol level) or non-invasive physiological data (e.g., weight, blood pressure, heart rate, body assessment data) can be collected. Food data based on the questionnaire can be collected either regularly or on an alternating daily or weekly basis. Baseline data (medical background, habit, preference, personal knowledge, initial weight and height, initial blood pressure, and initial body assessment data) are being collected for demographic statistics or population clustering or individual target assessment during the participant’s initial recruitment or every month.

**Figure 1:**
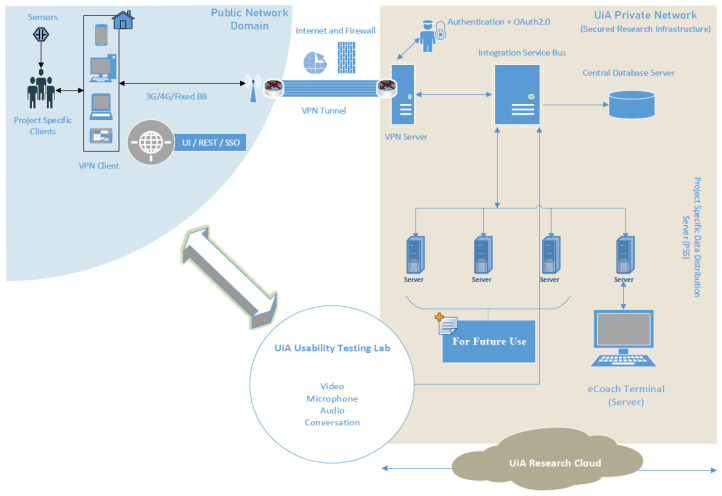
The developed digital infrastructure with extended SSK features.

**Figure 2:**
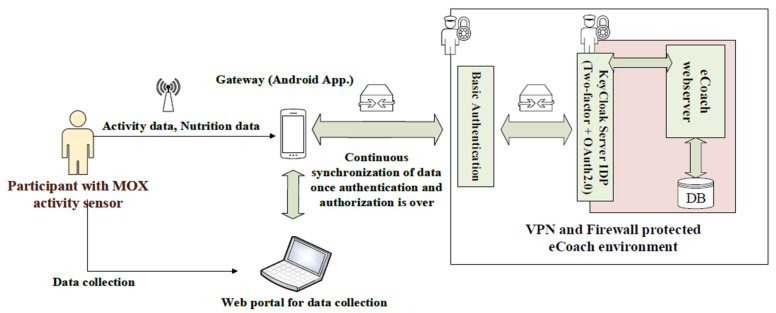
Deployment of eCoach system in the developed digital health infrastructure with extended SSK features.

In this context, we developed a digital infrastructure after extending the SSK features with VPN, Bcrypt hash, API key, firewall, and SSL, as depicted in Figure 1. Moreover, we deployed an electronic health coaching (eCoach) prototype system in the developed infrastructure to execute a formal security testing of the SSK solution scheme, and it is depicted in Figure 2. We maintained a modular structure for our eCoach prototype system for an obesity case study with the following modules [25]: activity, contextual, questionnaire, user interface (eCoachUX), and eCoach business (for user management, performance monitoring, database management, scheduling, user support, user communication, decision support, and recommendation generation).

### 3.1. Data Collection

We used a wearable MOX-2 [38] activity monitor to collect personal activity data for the following measurement parameters:

- Physical activity classification (low intensity, medium intensity, and high intensity),
- Posture detection (sedentary, standing, and weight-bearing),
- Physical activity intensity (counts per minute),
- The activity module is responsible for activity device registration, device allocation, seamless collection of sensor observations, and sending it back to the eCoach business module for storing data in a PostgreSQL database.

MOX-2 is an activity monitor based on an embedded BLE accelerometer with low power consumption. The device can seamlessly measure and transmit high-resolution raw acceleration data and multiple activity parameters per second for seven consecutive days (up to 60 days).

**Figure 3:**
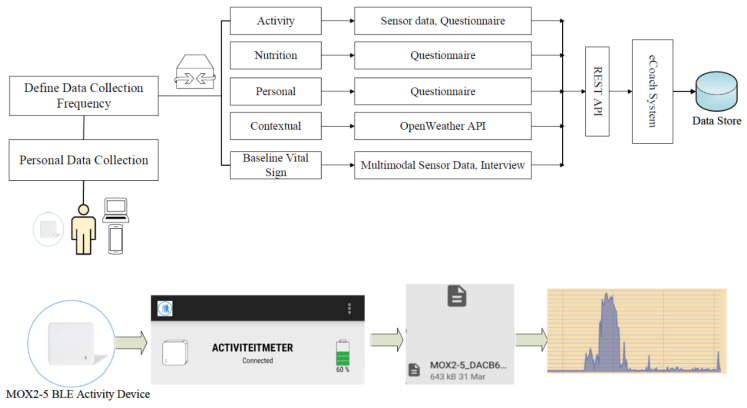
Data collection modules of the health eCoach prototype system.

In Stage 1, data collected from the activity sensor are sent back to the MOX mobile app to be stored temporarily in CSV format on the smartphone over BLE protocol. In Stage 2, a schedular running at the backend of our eCoach app collects activity data periodically from the smartphone location and sends it to the activity module using the HTTP-POST service (see Figure 3). The contextual module periodically contains context updates from OpenWeather REST APIs (e.g., latest and hourly) with API-Key authentication and sends them back to eCoach business logic for stable storage. The questionnaire module consists of six question sets: daily, alternative day, weekly, interview, baseline (monthly), and feedback form. The participant submits the questionnaire, which is stored in the database through the eCoach business logic.

### 3.2. eCoach System (App. Version vs. Web Version)

The “/eCoachUX/home” API is exposed to the external user (protected with VPN access, a firewall, and SSL). Other APIs are protected with the access-role as configured in the Keycloak authorization server. If participants forget the password for authentication, they need to raise a request through “/eCoachUX/complaint” REST API. The Actuator provides secure endpoints for controlling, handling, and monitoring Spring Boot modules, such as /metrics, /env, /beans, /health, /info, and /trace, which are protected by role-based authorization. The user interface module is responsible for app view, web view, and data visualization based on individual access roles. Figure 1 depicts how the SSK security solution for the eCoach system has been implemented with the KeyCloak third-party IAM platform. This study concentrated only on the eCoach API security and its implementation with SSK. Other core eCoach concepts, such as sensor specification, decision support principles, AI incorporation for the analysis of EHRs, data visualization, and personalized recommendation generation for a healthy lifestyle, are beyond this study’s scope.

The eCoach system has been hosted in a VPN-protected ubuntu infrastructure provided by The University, and the provided network (“EduNet”) is strictly firewall protected. Its internal IP addresses are not published to external Domain Name Services (DNS). Networks inside EduNet should be accessible; however, they must go through the proxy for external access from the eCoach server. We implemented an extra layer of basic (form-based) authentication on top of KeyCloak’s two-factor (password and One-Time Password (OTP)) authentication to authenticate participants on the eCoach mobile app to transfer activity data from the MOX-activity smartphone app. Basic authentication consists of a system-generated unique user ID (UUID) and a modifiable password. No personal data (such as national id, email, mobile or phone no, or similar personal identifiers) are unveiled in the basic user authentication step. KeyCloak’s two-factor user authentications consist of google authenticator (auto-id generator app) and credentials. Collected data are stored in a PostgreSQL database in JSON format for faster data processing. Furthermore, we created a self-signed SSL certificate with Keytool to secure confidential web information using public key (RSA) encryption. The eCoach system has five user categories: researcher, developer, system admin, health professional (nurse), and participants. They are further grouped into “ADMIN” (researcher, developer, system admin) and “USER” (health professional or nurse, and participants) for role-specific access control. Researchers and developers are responsible for the feasibility study, methodology adoption, system design, development, system configuration, deployment, test, and performance evaluation. They have full access to the system. System admin is accountable for infrastructure support. However, they do not have access to the participant’s health and wellness data. Trained health professionals, such as nurses, are responsible for interviewing (participant screening, recruitment, and the assessment of health condition), thereby collecting initial and baseline data through a pre-defined questionnaire set. Furthermore, they have access to a visualizing dashboard to monitor the participant’s processed health and wellness data. Participants have access to the personal data collection endpoints, feedback forms, and personal health and wellness monitoring dashboard. The system is protected from disclosing any personal data, and questionnaire forms are restricted from submitting any unique identifiers. The Secure Shell (SSH) access to the ubuntu and database servers is protected with authentication and authorization rules. The proposed eCoach system can be accessed through a web portal and/or a smartphone android application.

### 3.3. Methods for Security Implementation and Performance Evaluation

There is no single protection method to meet all the security requirements and design specifications for our distributed eCoach system. To implement and validate the SSK security solution, we utilized the web engineering security methodology by Aljawarneh et al. [39]. The software engineering principles inspire the method and build on top of the standard waterfall software development life cycle (SDLC) (see Table 3). The methodology helped to eliminate substantial threat exposures during all the SDLC phases by integrating security and evaluation components at each SDLC phase. Both software engineers and security professionals verified each stage.

To determine the performance of the hybrid security method, we evaluated the scalability of the API. Throughput (S) and latency (L) are considered to measure API scalability [39,40,41,42]. Network throughput refers to the average data rate at which data or messages are successfully transmitted on a specific communication link. It is measured in bits per second (bps). The maximum network throughput equals the Transmission Control Protocol (TCP) window size divided by the communication packet’s round-trip time (RTT). This method does not consider communication overhead, such as network receiver window size, machine limitations, or network delay [39,40,41,42]. Network latency is the time it takes for a packet to be captured, transmitted, processed through multiple devices, and then received and decoded at the destination [39,40,41,42]. We use Apache open-source software JMeter (V 5.4.1) to generate HTTP request load (l) to check API scalability as a “thread group” and capture the corresponding throughput and latency. The following three attributes for load testing using JMeter [39] are critical:

- The number of threads or users;
- The acceleration period in seconds;
- The loop count sets the test count.

We set the cycle count value of a single load to 5 repeated experiments and take the average throughput and latency. Low latency and high throughput are good performance indicators for supporting real-time critical applications.

## 4. Adopted Security Scheme

This section describes how we adopted the security method for our security implementation with SSF and Keycloak and, afterward, the solution validation. Then, we describe security configuration, password management, and security testing to check the SSK security solution’s effectiveness to protect MSA APIs only. We tested the security performance of the system in both real-time and simulated environments.

### 4.1. Hybrid Security Scheme—SSK

In this study, we built a Spring Boot application and integrated it with Keycloak [27] to protect the REST APIs from unauthorized calls. We created users in Keycloak, login and generated a JWT token [43] to access the secured REST APIs. We configured the KeyCloak server with the following steps: a. download and run the KeyCloak server in stand-alone mode; b. configure the server with the master realm, new eCoach specific realm, login configuration, email settings, theme and internationalization, creation and management of clients, and realm level roles; c. add Keycloak Spring maven dependencies (keycloak-spring-boot-starter) and configuration of respective key-value pairs; and d. create and configure Java class with the Spring security (@EnableWebSecurity), Spring Security global method security (@EnableGlobalMethodSecurity), and extension of Keycloak web security configuring adapter class.

**Figure 4:**
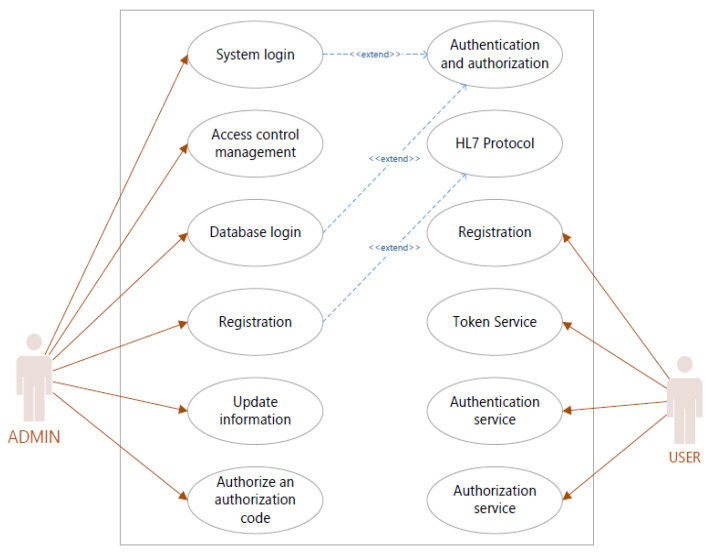
Authorization Server (AS) use-case for eCoach prototype system.

**Figure 5:**
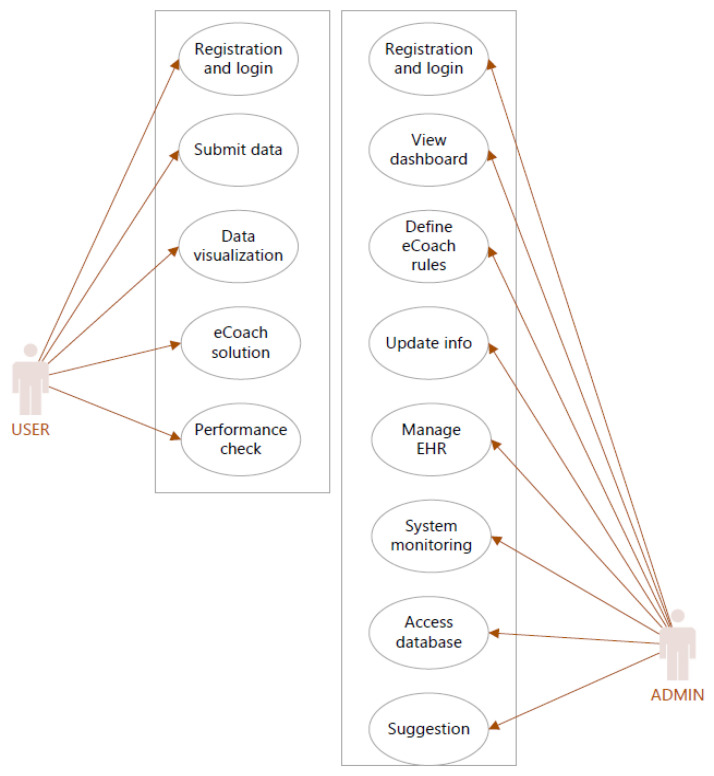
Resource Server (RS) use-case for eCoach prototype system.

Clients are services and applications that can request the authentication of users through Keycloak. There are two types of clients [27]. The first kind of client is Keycloak applications that want to encrypt themselves and use SSO. The second category of clients are applications that request an access token to use that access token to access protected resources. Client ID is used in the request to identify the client. We used OpenID Connect (OIDC) as a client protocol in KeyCloak to secure eCoach APIs. There are three access types [27]: public, confidential, and bearer-only under the OIDC client to grant access. Here, we set access type confidential to obtain client secret and turned-on features, such as Standard Flow Enabled, Direct Access Grants Enabled, Service Accounts Enabled, Authorization Enabled options [27]. Confidential access type is used for server-side clients to perform login using a client secret and request access tokens to access resources. A service that can authenticate a user is an identity provider (IDP). Keycloak is an IDP [27] and acts as an authorization server (AS) (@EnableAuthorizationServer) in the OAuth2 workflow. An authorization point works on the AS, allowing our applications and HTTP endpoints to define our system’s features. In our eCoach system, two actors who interact with the AS are—ADMIN (resource owner) and USER (client registered with AS) as the fundamental use cases depicted in Figure 4. The resource server (@EnableResourceServer) is an application that provides clients with an access token to access the HTTP endpoint resource server. It is a library set that includes HTTP endpoints, static tools, and interactive web pages. Our implemented microservices are depicted in Figure 5 as use cases of resource servers. OAuth2 is a mechanism for authorization to allow access to the client resources. We focused on the grant form (authorization code), client ID, and client secret to creating an OAuth2 application.

**Figure 6:**
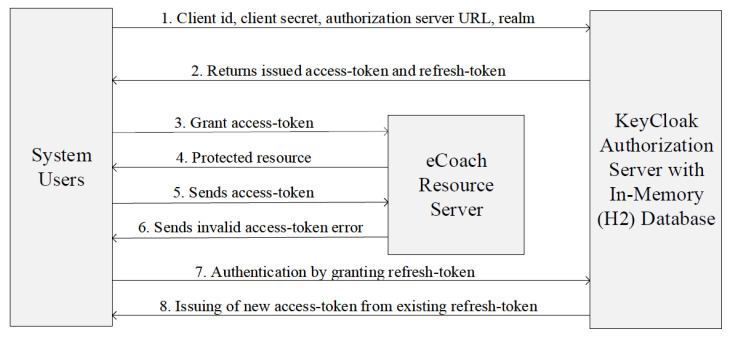
Access-token generation from the existing refresh-token.

JavaScript Object Notation Web Token (JWT) represents the claims between two parties in a JSON Web Token [14,15,16]. Such tokens are of two types: identity token (part of the OpenID Connect specification that is a client-dedicated function namespace) and access token (part of the OpenID Connect and OAuth2 specification and allows HTTP request that grants access to the service). We used ES256 to create a JWT signature. Access tokens are typically short-lived and frequently expire after only minutes. The additional refresh token sent by the login protocol allows a new access token to be accessed by the application after it expires (see Figure 6). Our Spring application security configuration expands the KeyCloak’s built-in KeycloakWebSecurityConfigurerAdapter, to include the following features: configure and configureGlobal methods with HttpSecurity to protect application APIs from external attack, authorization, and authentication of an HTTP request, password management with the pbkdf2-sha256 algorithm, session authentication strategy, filter registration, and Keycloak-Springboot configuration resolver. eCoach’s OAuth configuration extends the core classes of the KeyCloak library in SSF for user creation, authentication, and retrieval of role-specific access tokens based on authorization server Uniform Resource Locator (URL), realm, client ID, role, and client secret. The user details and their tokens are stored in the in-memory H2 database [27].

### 4.2. Developing SSK in an Architecture

We deployed the SSK security scheme in our digital architecture with extended security features (see Figure 2). Cross-Origin Resource Sharing (CORS) is a protection principle that enables resources implemented in web browsers to be restricted. It keeps the external code from creating or consuming requests from unwanted sources. Our eCoach’s RESTful APIs support the CORS on SSL-enabled tomcat port 8443 with @CrossOrigin annotation. By design, the Spring Boot application uses the HTTP 8080 port when the application begins. However, we created a self-signed SSL certificate to enable HTTPS and port 8443 with the code—keytool-genkey-alias apachetomcat-storetype PKCS12-keyalg RSA-keysize 2048-keystore eCoachKey.p12-validity 3650. The Keystore file path was then added to the configuration file of the Apache-tomcat webserver to change the application startup port from 8080 to 8443. Port 8443 is the default configuration in the Apache-tomcat webserver to allow HTTPS traffic. Moreover, the port number can be customized.

Passay [44] is a password generation and validation library based on the Java programming language. It offers a comprehensive list of features for validating/generating passwords and is highly configurable. Its API has the following three core components: Rule (defines password generation policy), PasswordGenerator (password generation with defined ruleset), and PasswordValidator (validates password against a defined rule). We used Passay to generate an initial (system-generated) alphanumeric password of 10-letters and Bcrypt [45] for form-based authentication on the eCoach mobile application to upload activity data from the mobile to the eCoach server. In the sign-up process, the user will enter email, mobile, and role as input, and the system will create their UUID, default encrypted password for basic and Keycloak authentication. The user must change their default password after successful account creation and enable the google authenticator on their mobile phone for 6-digit OTP generation with the SHA512 algorithm for the two-factor authentication process at Keycloak.

**Figure 7:**
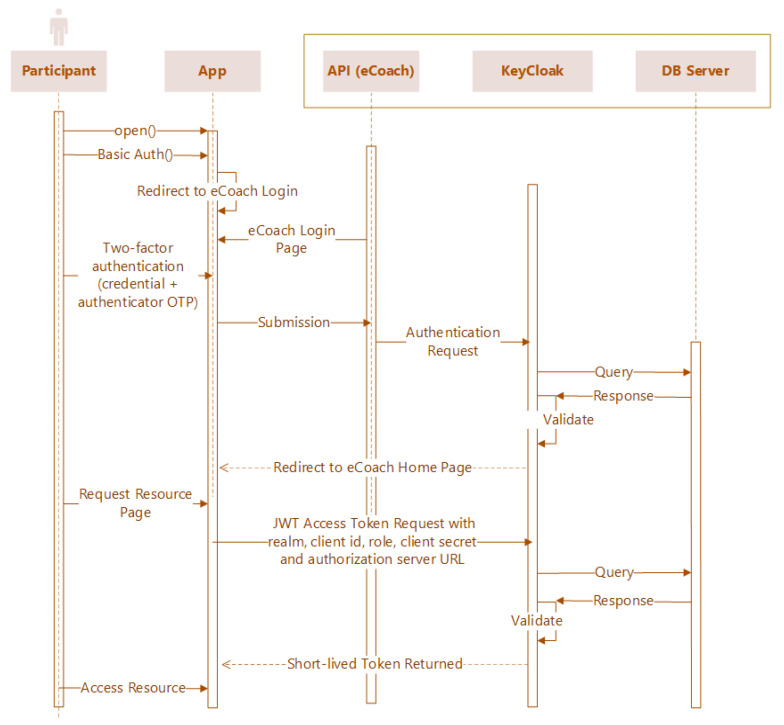
Sequence diagram of the login process for accessing web resources following the SSK security solution.

Figure 7 illustrates the sequence diagram of the SSK-based authentication and authorization process flow on the eCoach prototype system. The scoped software, libraries, respective versions, and their purpose of usage are described in Table 4 and Table 5.

## 5. Experimental Results and Discussion

### 5.1. Experimental Setup

A security testing method is designed to expose weaknesses in an information system’s security mechanisms that safeguard data and preserve functionality as expected. It can normally be broken down into measures that are functional and non-functional [46]. Security checks are carried out in many ways, each of which is intended to verify the security principles [46,47]. We used unit testing and non-functional penetration (pen) testing as security testing methods. Unit testing was performed with Postman, web-browser, and Mock MVC (Mockito) [48] to check security intended functionalities. Penetration testing facilitates finding out the weaknesses of a computer system, a web application, or a network. It can be of three types—black box testing, white box testing, and gray box testing. The most common penetration testing method is to scan the target for vulnerabilities. The target of penetration testing can be an operating system, database system, application, or network environment. This study has focused on the security of the MSA APIs and concentrated on penetration testing for network environments. Therefore, we used Wireshark software only for gray box penetration testing for web application tests that checked the security vulnerabilities (e.g., DoS, DDoS, IP spoofing, port scan) at the network side.

We implemented the SSK security solution first in an unprotected windows environment. We then deployed the codebase in a Linux environment (see Table 6), protected with VPN and network firewall to compare pen testing’s [47,48] performance in two scenarios. In addition, we set up Apache JMeter to perform a scalability testing of the adopted approach.

### 5.2. Experimental Results

All the simulated attacking parameters, outcomes, and network protocol analysis report results are described and discussed in this section. Experiments related to CSRF, XSS, Clickjacking, content sniffing, and brute force were conducted with Mockito, Keycloak UI, and Postman. DoS, DDoS, IP spoofing, MITM, and port scanning were performed with simulated code, Wireshark network analyzer, based on explicit filter patterns (see Table 7), system commands in Windows and Linux (Ubuntu) environments. In the Spring codebase, we created eight unit-test cases with Mockito for test user creation with role assignment, basic (HTTP form-based) user authentication, two-factor user authentication (password + OTP), and role-based authorization (OAuth2) with an access token. We executed a negative test scenario where the user received an expected error response code (*HTTP 401*) due to prohibited access to an unauthorized resource API endpoint. Table 8 defines the combined result of Mockito test performance in a vanilla test setting where we compared our API response time with preferred, acceptable, and delayed response time. The response metrics can be classified into the following categories—mean response time, peak response time, and error rate. We have considered the mean response time in this study.

Spring boot application extends default spring security class WebSecurityConfig to allow protection against CSRF attacks. CSRF tokens are powerful and unpredictable, created as session tokens with specific properties that the attacker cannot calculate or predict. Both post forms in the “Java Server Page (JSP)” or template files need to include the CSRF token. If it is a JSON call, the token must be added to the header. Initially, we disabled KeyCloak token-based security setup and extended Spring’s default web security configuration. Subsequently, we executed the following four test cases for CSRF attack with Postman—CSRF disabled (valid credential, invalid credential), and CSRF enabled (valid credential and valid _csrf token, valid credential, and invalid _csrf token). Successful authentication resulted in an HTTP status code 200 or 201. However, we disabled the CSRF token generation in actual security solution implementation. The reason for disabling CSRF is that our developed spring boot application is available to the public. Therefore, we replicated similar and more robust web security measures with KeyCloak’s two-factor authentication and access token-based authorization. The successful test results are captured in Table 9. To ensure protection against XSS, Clickjacking, and content sniffing, we facilitated KeyCloak’s configurable security defense. We tested the attack with a client’s HTTP POST request with Postman for new user creation and investigated whether XSS, Clickjacking, and content sniffing securities are allowed in the response header. Setup data and documented response headers as shown in Table 9.

To ensure protection against brute force attacks, we enabled KeyCloak’s configurable security defense. According to our Keycloak security configuration, the user will get a chance of a maximum of six login failures before their account gets locked. The failure reset time is 12 h. Our analysis uses Spring Security with KeyCloak to model the case attacker using the password dictionary to execute the brute force attack. We set up the data in Postman and recorded the response headers. The brute force attack’s unit test with Postman is presented in Table 10.

We first tested the DoS and DDoS attack in an unprotected windows server, where we configured the KeyCloak server and Apache-tomcat web server to deploy the eCoach prototype system. Then, we wrote a Java multi-threading code [49] to create parallel HTTP calls to an example exposed API. For checking the DoS attack, we executed the java code from a single JVM, and for simulating DDoS, we executed the code from multiple JVMs in parallel. After an average of 3–5 min of parallel URL connection request traffic, the server produced high utilization of CPU and memory high network throughput, and thus, resulting in unresponsive API.

We used tcp.flags.syn == 1 and tcp.flags.ack == 1 and tcp.flags.syn == 1 and tcp.flags.ack == 0 filter in Wireshark to detect TCP SYN floods due to DoS attack and found how the windows server IP was flooded with incoming packets. We replicated the experiment in the Linux server protected with VPN and network firewall. We observed that packets outside of the VPN were blocked. We utilized other filters specified in Table 7 to analyze incoming network packets to detect attacks, such as IP spoofing and MITM with Wireshark. To detect network packet sniffing with Wireshark, we studied source and destination IP, ports, and protocols, such as Media Access Control (MAC), Dynamic Host Configuration Protocol (DHCP), DNS, TCP, User Datagram Protocol (UDP), and Address Resolution Protocol (ARP) with the following metrics: packet count, rate (milliseconds or Milli sec.), percent, burst rate, and burst start. We found that VPN protects the eCoach E-2-E network communication. A network firewall, and an SSL certificate on top of the API endpoint access security, made the eCoach API endpoint safe from major external vulnerabilities, such as DoS, DDoS, IP spoofing, and MITM. API endpoint security indirectly ensures the privacy of personal health data inside of our eCoach prototype system.

**Figure 8:**
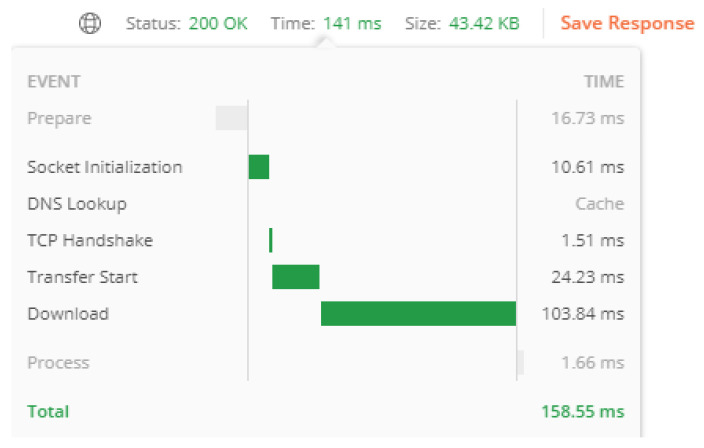
Break-up of a response time in our proposed solution for a single authorized HTTP request.

To perform scalability testing in JMeter, we selected an eCoach REST service with an approximated 257 Bytes of a request body, 43.42 Kilobytes of the response body, and a response time of 141 msec. (see Figure 8). Using JMeter “Thread Group” feature, concurrent threads, or loads (X) had been created with three different values of ramp-up seconds (Y) and a loop count value of five (Z). At each iteration, X × Z number of loads were created to capture mean throughput and mean latency time. The results are described in Table 11, Table 12 and Table 13. The result shows a direct proportion between throughput and load (S α l) and latency time and load (L α l). However, achieving a certain threshold, the throughput sinks with increased load (S α $\frac{1}{l}$). We have considered the following values for scalability testing; however, the range can be increased for the upcoming studies.

X = {1, 10, 25, 50, 75, 100, 200, 300, 500}

Y = {1, 5, 10}

Z = {5}

## 6. Discussion

Using the SSK security solution, personal health data governance has fulfilled the General Data Protection Regulation (GDPR) compliance checklist as specified in Table 14. It is three-fold research:

- First, we implemented a security solution with SSF, basic authentication, two-factor authentication, and authorization (OAuth2) with an open-source KeyCloak server (IDP), VPN, network firewall, and SSL.
- Second, we implemented the solution for developing a digital infrastructure where we deployed an eCoach prototype system.
- Third, we performed testing of the prototype APIs against the common external vulnerabilities, as described in Table 15.

We created 22 test cases for 16 test scenarios to replicate external attacks as our SSK security solution. SSK security implementation and configuration in the prototype system successfully secured the eCoach APIs from an attack in all scenarios with 100% test accuracy. Furthermore, we performed a qualitative analysis on the effectiveness of SSK in Table 16 after comparing SSK with Spring Security and Keycloak against certain security features.

Due to licensing and subscription constraints, we could not create a similar environment and deploy other solutions (e.g., SF + Okta or solutions identified in literature) to test scalability against throughput and latency. Therefore, we only performed scalability testing for our adopted work under defined settings and obtained a promising result, as described in Table 11, Table 12 and Table 13. The result shows that the increased value of the ramp-up period has a positive impact on the mean throughput and mean latency. Moreover, we performed a comparative analysis of our adopted security solution with existing MSSA as described in Table 17 and Table 18. The solution is safe from the typical external illegitimate flooding requests as the external or exposed eCoach services are protected by a VPN and a firewall.

This study has consolidated the security implementation with Keycloak third-party IAM combined with SF and its performance evaluation in digital infrastructure. In future research, we will connect other third-party IAM (e.g., Okta, TSD) with SF to implement different security solutions and compare their performance evaluation against SSK. This study strictly focuses on SSK implementation and its performance analysis in an “on-premises” digital setup. In the future, we will extend our study in a cloud setup for SSK solution implementation, performance analysis, and comparing the performance outcome with the “on-premises” SSK performance results.

## 7. Conclusions

The literature review reveals that existing frameworks and security standards to secure the API endpoints in an MSA architecture have minimal experimental outcomes of confirming the integrated effectiveness of SF, SSF, third-party OAuth2, Bcrypt hash, VPN, firewall, and SSL. A prototype eCoach system has been implemented in this study using MSA as a prototype to assess the integration between the technologies. The research findings show that the SSK solution effectively protected the APIs from the vulnerabilities, such as CSRF, XSS, Clickjacking, content sniffing, brute force, DoS, DDoS, IP spoofing, and MITM. Integration of SSF with Keycloak has made the solution powerful and highly customizable. The overall solution is scalable, with approximately 300 concurrent requests. We can extend the study with other different penetration testing methods in the future.

## Figures and Tables

**Table 1:** Selected list of core security components of Spring Security Framework.

Components	Description
SecurityContextHolder	It gives access to the SecurityContext.
SecurityContext	It contains the Authentication class and request-specific security information.
Authentication	This class stores user information for representing the principal in a way unique to Spring Security.
GrantedAuthority	The application-wide permissions given to a principal are to be reflected with this class.
UserDetails	It gives necessary information to create an authentication object from DAOs or other security data sources.
UserDetailsService	It helps to build UserDetails when a String-based username is passed (or certificate ID or similar).
AuthenticationManager	It loads the user authentication data (credentials or user store’s information) to verify authenticity of the users.
AuthenticationManagerBuilder	It is used to set up user information in memory, Java Database Connection (JDBC), Lightweight Directory Access Protocol (LDAP), or adding a custom UserDetailsService.
AbstractSecurityInterceptor	A central class of authorization helps to intercept secured resource access.
SecurityMetedataSource	It provides details about the current user and the item being protected along with SecurityContext class.
AccessDecisionManager	To decide dynamically if access can be granted to a user.
AbstractSecurityInterceptor	To perform access decisions.
WebSecurityConfig	It enables http security to access the HTTP Endpoints with basic authentication by extending WebSecurityConfigurerAdapter and overriding configure method.
PasswordEncoder	The PasswordEncoder interface of Spring Security is used to convert a password in a single way to allow the password to be securely stored. It can use any of the following encryption algorithms—MD5, SHA-256, and Bcrypt (recommended).
AuthenticationProvider	It helps to protect application with Spring Security and Basic Auth.
CorsRegistry	To set up global support for CORS configuration for the Spring Boot application.
HttpSecurity	It is like the Extensible Markup Language (XML) <http> feature of Spring Protection in the namespace configuration. It enables web-based authentication for individual http requests to be configured. It will apply to all requests by default.

**Table 2:** Selected list of Keycloak components for spring security framework integration.

Components	Description
KeycloakWebSecurity ConfigurerAdapter	As a convenient base class, Keycloak provides a KeycloakWebSecurityConfigurerAdapter to build a WebSecurityConfigurer case. The execution enables customization by overriding techniques. It greatly simplifies context configuration for security.
KeycloakAuthenticationProvider	On a KeycloakAuthenticationToken, it performs authentication.
EnableGlobalMethodSecurity	The jsr250Enabled property allows the annotation of @RoleAllowed to be used.
KeycloakConfigResolver	It resolves the configuration of Keycloak Spring Boot Adaptor using “keycloak.json” configuration file.
sessionAuthenticationStrategy	This method defines the session authentication strategy.
RolesAllowed	This annotation is the JSR-250′s equivalent annotation of the @Secured annotation.

**Table 3:** Importance of the SDLC processes in eCoach context.

Process	Importance in eCoach context
Requirement Specification	Defining of eCoach scope, eCoach target audiences, user needs, functional requirements, external interface requirements (user, hardware, software, communications), system features, authentication, and authorization requirements to handle internal and/or external attacks, requirements for data integrity, and storage, and other non-functional requirements (performance, safety, security, and quality).
Design	Sufficient attention to role creation, data federation, password management, system modulation, API design, security configuration to integrate SSF with KeyCloak, UML modeling, database design, server configuration, and consideration for security vulnerabilities.
Implementation (development)	Development of APIs using Spring Boot Framework, coding for UI design, data collection, data storage, password management, authentication, and authorization.
Functional Testing (unit testing)	In two levels, which are unit testing (unit testing) and non-functional penetration testing, we break down security testing of the eCoach system.
Maintenance	This phase involves bug fixing, infrastructure support, support to participants, addition/deletion of users, and upgradation.

**Table 4:** Scoped software, respective version, and usage.

Software	Version	Purpose
Spring Boot Framework	2.5.x	A framework for system development following the design pattern
Java compiler	JDK 15.x+	To compile java codes
Mongo DB	5.0.2	To store and query PGDs
Mavens build tool	3.8.2	To build application and resolve dependencies
Spring Tool Suite	4.7.0	To Code in Java programming language
KeyCloak	13.x	To work as identity provider server
Java Passay	1.6.1	To create initial systems generated password
Mockito	3.12.x	To perform unit testing
Apache Tomcat	10.x	To deploy eCoach modules
Microsoft Visio	Office16	For UML modeling and drawing
Notepad ++	7.8.4	For editing text, viewing log, and html coding
Bootstrap, Thymeleaf	4.x	User interface design with HTML5 and CSS
Wireshark	3.4.8	To analyze network traffic or packets
Postman	9.0.7	To Perform manual testing for REST APIs
JMeter	5.4.1	To perform API scalability testing
Apache Log4j	2.17.1	To perform logging

**Table 5:** Scoped libraries and respective version.

groupId	artifactId	Version
org.springframework.boot	spring-boot-starter-parent	2.5.x
org.springframework.boot	spring-boot-starter-security	
org.springframework.boot	spring-boot-starter-web	
org.springframework.boot	spring-boot-devtools	
org.springframework.boot	spring-boot-starter-test	
org.springframework.security	spring-security-test	
org.keycloak	keycloak-spring-boot-starter	13.x
org.keycloak	keycloak-admin-client	13.x
org.bouncycastle	bcprov-jdk15on	1.69
org.springframework.boot	spring-boot-maven-plugin	
com.google.code.gson	gson	2.8.7
org.apache.commons	commons-csv	1.8
org.apache.maven.plugins	maven-compiler-plugin	3.5.1
org.apache.maven.plugins	maven-project-info-reports-plugin	2.5.2
commons-lang	commons-lang	2.2
org.apache.commons	commons-lang3	3.9
org.springframework.boot	spring-boot-starter-thymeleaf	
org.springframework.boot	spring-boot-starter-mail	
com.twilio.sdk	twilio	7.16.1
javax.servlet	jstl	1.2
org.apache.tomcat	tomcat	10.0.11
org.springframework.boot	spring-boot-starter-actuator	
org.mockito	mockito-core	3.12.4

**Table 6:** Specification of the experimental environment.

Specification	Windows System	Linux System
Memory	8 GB	15 GB
Operating System	Windows 10	GNU/Linux
Disk (HDD)	235 GB	1023.9 GB
Socket endpoint	127.0.0.1:8081 (localhost)	10.225.147.186:8443 (Class A private IPV4)

**Table 7:** Explicit filter pattern to analyze network traffic.

Filter	Purpose
ip.addr == xxxx/ip.dst == xxxx/ip.src == xxxx	To authorize API access based on bearer token
tcp.port == xxx/tcp.flags.reset == 1/tcp.stream eq X/tcp.seq == x/tcp.flags.push == 1/http.request/!(arp or icmp or dns)/(arp or icmp or dns)/udp contains xx:xx:xx/dns.flags.rcode ! = 0/http or dns/host xxx and not (port xx or port xx)/not broadcast and not multicast/broadcast and multicast/net xxx/port xx/ip.addr == x.x.x.x && ip.addr == x.x.x.x/tcp.stream eq xx/tcp.flags == 0x012/tcp.time_delta > .xx/tcp.analysis.flags && !tcp.analysis.window_update/ tcp dst port xx/ip.src ! = xxxx or ip.dst ! = xxxx	To define MIME type (ex. application/json)
dst port 135 and tcp port 135 and ip[2:2] == 48	To define the length of the request body
icmp[icmptype] == icmp-echo and ip[2:2] == 92 and icmp[8:4] == 0xAAAAAAAA	To define domain name for which the request is being sent
udp.srcport == 53 or udp.srcport == 123	To define the form of response content type
tcp.flags.syn == 1 and tcp.flags.ack == 0	To define compression algorithm as response
tcp.flags.syn == 1 and tcp.flags.ack == 1	To keep underlying network connection (e.g., alive or close)

**Table 8:** Performance of unit-testing with Mockito framework.

Scenario	Input	Mean Response	Category		
			Preferred Response Time (0.1 s)	Acceptable Response Time (<2 s)	Delayed Response Time (10 s)
User creation with a valid role	Email, mobile, role	1–2 s	Yes	Yes	No
Retrieval of access token and refresh token	ClientID, client secret, grant type, UUID, password	0.1–1 s	Yes	Yes	No
HTTP basic authentication	UUID, Password	0.01–0.03 s	Yes	Yes	No
KeyCloak two-factor authentication	UUID, Password + OTP	0.1–1 s	Yes	Yes	No
Authorized access	Valid access token	0.1–1 s	Yes	Yes	No
Unauthorized access	Invalid access token	0.01–0.05 s	Yes	Yes	No

**Table 9:** Mockito unit testing performance.

Scenario	Parameters	Value
Input Parameters for HTTP POST	Endpoint	/eCoachUX/createParticipant
	Port	8443
	HTTP Verb	POST
	Information	Email, mobile, role
Data collection—1	Response Header: X-XSS-Protection	1
	Response Header: mode	block
Data collection—2	Response Header: X-Content-Type-Options	nosniff
Data collection—3	Response Header: X-Frame-Options	DENY

**Table 10:** Brute force attack’s unit testing with Postman.

Scenario	Comment
Normal successful login	No brute force detected
Normal login failure (< 6 times continuously)	No brute force detected
Normal login failure (6 times continuously)	Exceeded maximum attempts allowed. Brute force attack detected and account locked.

**Table 11:** Scalability testing results with Y = 1, Z = 5, and variable loads (X).

Y = 1, Z = 5	Mean Throughput	Error %	Received KB/s	Delivered KB/s	Mean Latency (s)
Load (X)					
1	6.4	0	265.5	6.8	165
10	34.7	0	1496	5.1	112
25	55.7	0	2410	8.04	260
50	78.5	0	3398.5	11.35	440
75	95.3	0	4130.5	13.77	550
100	112.8	0	4891	16.27	674
200	104.5	0	4529	15.1	1597
300	169.3	0	7347.3	24.46	1365
500	139.5	0	6048	20.4	2900

**Table 12:** Scalability testing results with Y = 5, Z = 5, and variable loads (X).

Y = 5, Z = 5	Mean Throughput	Error %	Received KB/s	Delivered KB/s	Mean Latency (s)
Load (X)					
1	18.7	0	799.39	2.9	55
10	10	0	423.5	1.45	39
25	24.6	0	1062.33	3.8	24
50	49.2	0	2135.3	7.4	23
75	73.6	0	3194.8	10.65	24
100	98.2	0	4263	14.5	23
200	195	0	8479	28.5	26
300	258	0	11200	37.5	126
500	224	0	9729	32.6	796

**Table 13:** Scalability testing results with Y = 10, Z = 5, and variable loads (X).

Y = 10, Z = 5	Mean Throughput	Error %	Received KB/s	Delivered KB/s	Mean Latency (s)
Load (X)					
1	31.3	0	1358.5	4.8	34
10	6.5	0	279.5	1	24
25	12.5	0	537.5	2	25
50	25	0	1075	4	23
75	37.4	0	1611	5.8	23
100	49.6	0	2153.8	7.4	23
200	99.3	0	4307	14.6	23
300	148.6	0	6457	22	22
500	143.6	0	10586.5	35.5	47

**Table 14:** GDPR compliance checklist for SSK.

GDPR Checklist [50,51]	Addressed
Lawful basis and transparency	Yes
Data security	Yes
Accountability and governance	Yes
Privacy rights	Yes

**Table 15:** Summary of the adopted functional and non-functional testing.

Test Case Scenario	Test Case	Passed (Yes/No)
Basic authentication	Access with a valid credential	Yes
	Access with an invalid credential	Yes
Two-factor authentication	Access with a valid credential + OTP	Yes
	Access with an invalid credential/an incorrect OTP	Yes
New user creation and role assignment	Creation request with a valid role assignment	Yes
Role-based API access	Access with a valid access key	Yes
	Access with an invalid access key	Yes
CSRF disabled	Access with a valid credential	Yes
CSRF Enabled	Access with an invalid credential	Yes
	Access with a valid credential and valid “_csrf” token	Yes
	Access with a valid credential and invalid “_csrf” token	Yes
CSRF Disabled but Access Token enabled	KeyCloak-based authentication and authorization	Yes
XSS Attack	Validate with response header if XSS attack protection is enabled	Yes
Brute Force Attack	General login failure	Yes
	Multiple (=6) login failure	Yes
Content sniffing	Validate with response header if attack protection is enabled	Yes
DoS Attack	Analysis of network statistics and packet information	Yes
DDoS Attack	Analysis of network statistics and packet information	Yes
MITM Attack (sniffing)	Enabling HTTPS (SSL) and the analysis of network statistics	Yes
IP Spoofing	Analysis of network IP statistics and packet information	Yes
Port Scanning	Analysis of network port statistics and packet information	Yes
Clickjacking Attack	Validate with response header if attack protection is enabled	Yes
Total Test Pass Rate	-	100%

**Table 16:** Qualitative analysis on the effectiveness of SSK with three flags—No (0), Limited (1), and Yes (2).

Features	Spring Security	KeyCloak	SSK
OAuth2	Yes	Yes	Yes
SAML2.0	No	Yes	Yes
OpenID	No	Yes	Yes
WebFlux	Yes	Yes	Yes
Access control	Yes	Yes	Yes
Identity and Access Management—Single-Sign-On (SSO), Identity Brokering and Login, User Federation, Client Adapters	No	Yes	Yes
Entire process of seamless calling the Keycloak Authorization Server from Spring-boot	Yes	No	Yes
Robustness	Limited	Limited	Yes
Powerful and customizable	Limited	Limited	Yes
Handling of Java EE security constraints	Limited	Limited	Yes
Multi-factor authentication	No	Yes	Yes
Easy to use	Yes	No	Yes
JSON web token	No	Yes	Yes

**Table 17:** Comparative analysis of our security solution with existing MSSA with respect to the key attributes of a secure web application architecture.

Research	Inter-Tier Authentication	Server-Side Validation	Secure Communication	Data Encryption	Logging
Chatterjee et al. (our work)	Yes	Yes	Yes	Yes	Yes
Salibindla et al.	No	No	No	No	No
Xie et al.	Yes	No	No	No	No
Nguyen et al.	Yes	Yes	No	No	No
Dikanski et al.	Yes	No	No	No	No
Aloufi et al.	No	No	No	No	No
Beer et al.	No	Yes	No	Yes	No
Serme et al.	No	No	No	Yes	No
Backere et al.	No	No	Yes	No	No

**Table 18:** Comparative analysis of our security solution with existing MSSA with respect to the implemented security features.

Research	Multi-Factor Authentication	OAuth2 (Token and Identity Brokering)	SSL/TLS	Bcrypt Hash	API Key	Spring Security	Third-party IAM (e.g., KeyCloak)	Protection against CSRF, XSS, Clickjacking, Content Sniffing, BF, DoS, DDoS, IP Spoofing, and MITM	CORS	Multi-Factor Authentication
Chatterjee et al. (our work)	Yes	Yes	Yes	Yes	Yes	Yes	Yes	Yes	Yes	Yes
Salibindla et al.	No	No	No	No	No	No	No	No	No	No
Xie et al.	No	Yes	No	No	No	Yes	No	No	No	No
Nguyen et al.	No	Yes	No	No	No	Yes	No	CSRF, BF, XSS	No	No
Dikanski et al.	No	Yes	No	No	No	Yes	No	No	No	No
Aloufi et al.	No	No	No	No	No	No	No	No	No	No
Beer et al.	No	No	No	No	No	No	No	DoS, DDoS, BF	No	No
Serme et al.	No	No	No	No	No	No	No	No	No	No
Backere et al.	No	No	TLS	No	No	No	No	No	No	No

## Data Availability

Not applicable.

## Abbreviations

APIs	Application Programming Interfaces
VPN	Virtual Private Network
SSL	Secure Socket Layer
eCoach	Electronic Coach
SF	Spring Framework
SSF	Spring Security Framework
DoS	Denial-of-Service
DDoS	Distributed Denial-of-Service
MITM	Man in the Middle
IP	Internet Protocol
OAuth	Open Authorization
OpenID	Open Identifier
CORS	Cross-Origin Resource Sharing
HTTPS	Hypertext Transfer Protocol Secure
RSA	Rivest–Shamir–Adleman
Bcrypt	Blowfish and crypt
SHA	Secure Hash Algorithm
MD5	Message-Digest algorithm
CSRF	Cross-Site Request Forgery
XSS	Cross-site scripting
IAM	Identity and access management
IoMT	Internet-of-Medical-Things
MSA	Microservice Architecture
PoC	Proof of Concept
MQTT	Message Queue Telemetry Transport
PKI	Public Key Infrastructure
TLS	Transport Layer Security
SSL	Secure Socket Layer
REST	Representational State Transfer
EHR	Electronic Health Record
SAML	Security Assertion Markup Language
JDBC	Java Database Connection
LDAP	Lightweight Directory Access Protocol
ACL	Access Control List
CAS	Central Authentication Service
SSE	Spring SAML Extension
SOAP	Simple Object Access Protocol
XML	Extensible Markup Language
OIDC	OpenID Connect
NSD	Norwegian Centre for Research Data
BLE	Bluetooth Short-Range Communication Technology
UUID	Unique User ID
DNS	Domain Name Service
OTP	One-Time Password
SSH	Secure Shell
SDLC	Software Development Life Cycle
BPS	Bits per Seconds
TCP	Transmission Control Protocol
RTT	Round Trip Time
IDP	Identity Provider
AS	Authorization Server
JWT	JavaScript Object Notation Web Token
JSON	JavaScript Object Notation
URL	Uniform Resource Locator
MAC	Media Access Control
DHCP	Dynamic Host Configuration Protocol
UDP	User Datagram Protocol
ARP	Address Resolution Protocol
GDPR	General Data Protection Regulation
FISMA	Federal Information Security Management Act
ISO	International Organization for Standardization
PCI DSS	Payment Card Industry Data Security Standard
JSP	Java Server Page
